# Drug-loaded nanoparticles reduced platelet aggregation and blood coagulation

**DOI:** 10.3389/fmedt.2025.1690389

**Published:** 2026-02-04

**Authors:** Sarah Majin, Afrida Malik, Pratima Poudel, E. Vincent S. Faustino, Nubia Zuverza-Mena, John Hwa, Seyedtaghi Takyar, Susan Shea, Kagya A. Amoako

**Affiliations:** 1 Department of Chemistry and Chemical & Biomedical Engineering, University of New Haven, West Haven, CT, United States; 2 Department of Pediatrics, Yale University, New Haven, CT, United States; 3 Analytical Chemistry, Connecticut Agricultural Experiment Station, New Haven, CT, United States; 4 Yale Cardiovascular Research Center and Yale Cooperative Center of Excellence in Hematology, Yale School of Medicine, New Haven, CT, United States; 5 Critical Care & Sleep Medicine, Yale University Pulmonary, New Haven, CT, United States; 6 Department of Surgery, University of Pittsburgh, Pittsburgh, PA, United States

**Keywords:** platelets, liposomes, nanoparticles, blood-contacting medical devices, thrombosis, drug delivery, nitric oxide, anti-coagulation

## Abstract

Surface-induced thrombosis remains a significant limitation of blood-contacting medical devices, driven primarily by platelet activation and rapid clot formation. Nitric oxide (NO), a potent endogenous antiplatelet agent, has therefore inspired the development of targeted NO-delivery strategies to mitigate device-associated thrombosis. In this study, nitric oxide-releasing antiplatelet lipid nanoparticles (anti-PLT LNPs) were formulated and characterized for their physicochemical properties and NO-release kinetics. Their platelet aggregation inhibition was evaluated in vitro, while mammalian cell biocompatibility and effects on whole-blood coagulation were evaluated using activated clotting time (ACT) measurements. Anti-PLT LNPs exhibited sustained and measurable NO release after 22 weeks of storage and demonstrated high mammalian cell biocompatibility. The nanoparticles inhibited platelet aggregation by up to 84.4% (*p* < 0.01) and significantly prolonged whole-blood clotting time, resulting in up to a threefold increase in ACT (*p* < 0.05). These findings indicate that NO-releasing anti-PLT LNPs effectively suppress platelet activation and coagulation while maintaining biocompatibility, offering a customizable and targeted approach to mitigate surface-induced thrombosis in blood-contacting medical devices.

## Graphical Abstract

**Figure:**
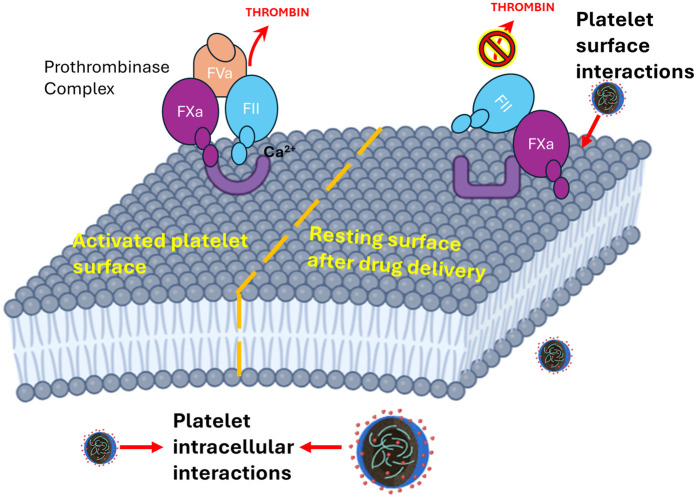
Modification of platelet function using nanomedicine. BioRendered.

## 1 Introduction

Surface-induced thrombosis remains a major challenge in medical devices such as extracorporeal membrane oxygenation (ECMO) circuits, stents, and artificial valves, where altered blood flow and contact with artificial surfaces (AS) initiate protein adsorption, platelet (PLT) activation, and coagulation (1, 2). These reactions increase the risk of device failure, occlusion, and embolization (3–5). Early in AS-induced thrombosis, factor XII (FXII) activation plays a central role, and its deficiency reduces thrombosis without increasing bleeding, making it a compelling therapeutic target (6–9). Clots on the AS form around activated platelets, which provide procoagulant surfaces and support fibrin formation (10–12). Although hydrophilic and zwitterionic coatings such as poly (carboxybetaine methacrylate) (pCBMA), poly (sulfobetaine methacrylate) (pSBMA), and poly (2-methacryloyloxyethyl phosphorylcholine) (pMPC) can reduce protein and platelet adsorption, their application to complex geometries such as tightly packed hollow-fiber oxygenators remains limited by incomplete surface coverage. Platelets are also activated by complement proteins, further amplifying AS thrombosis and emphasizing the value of interventions that act on platelet-driven pathways while minimizing effects on systemic coagulation (9, 13, 14).

Heparin remains the clinical standard for preventing device-associated clotting, acting by enhancing antithrombin III to inhibit factor Xa and thrombin. However, because it blocks both intrinsic (device-mediated) and extrinsic (tissue-mediated) pathways, heparin significantly increases bleeding risk (15, 16). Although selective intrinsic-pathway anticoagulants show promise (17–21), none are yet device-specific or clinically validated for patients reliant on extracorporeal technologies.

Precision drug delivery offers an alternative strategy by localizing antithrombotic activity while reducing systemic bleeding. Clot-targeted thrombin-cleavable nanoparticles (CTNPs) exemplify this approach by binding activated platelets and fibrin, carrying plasmin, and releasing them in response to thrombin (22, 23). These and other advanced drug-delivery systems (ADDSs), including nanocarriers and biomimetic vehicles, aim to improve antithrombotic pharmacokinetics and focus therapy at the thrombus site (23, 24), although translation to transient, device-specific thromboprophylaxis remains limited.

Nitric oxide (NO)–releasing nanomaterials offer another promising pathway. NO regulates vascular tone and inhibits platelet activation, yet its extremely short half-life necessitates controlled-release carriers such as polymeric or inorganic nanoparticles for cardiovascular applications (25). Macromolecular scaffolds are also under investigation to improve NO storage, stability, and targeted delivery (26).

In this study, we investigate liposomal nanoparticles formulated with clinically validated lipids, including those used in COVID-19 vaccines, to evaluate their ability to store and release NO, modulate platelet interactions, assess mammalian cell biocompatibility, and influence whole-blood coagulation. This work aims to establish an active, platelet-focused antithrombotic nanotechnology capable of precision targeting with reduced bleeding risk. Such an approach addresses critical gaps associated with current antithrombotic strategies, including the bleeding complications of systemic anticoagulants, limited validation of FXIIa inhibitors in device-bearing patients, and the instability or incomplete coverage offered by passive hydrophilic surface coatings.

## 2 Methods

### 2.1 Materials

1,2-Distearoyl-sn-glycero-3-phosphocholine (DSPC) and 1,2-Distearoyl-sn-glycero-3-phosphoethanolamine-N-[amino(polyethylene glycol-2000] maleimide (DSPE-PEG maleimide) lipids were purchased from Avanti lipids (Alabaster, AL, USA). Pooled male blood plasma was purchased from ZenBio (Durham, NC, USA), and centrifugations were done using a Beckman Coulter Allegra X-30R centrifuge (Indianapolis, IN, USA). Adenosine diphosphate (ADP), phosphate-buffered saline (PBS), and 1M hydrochloric acid were purchased from Sigma-Aldrich. Diazeniumdiolate dimethylhexane diamine (DMHD/N_2_O_2_) NONOate was used as a NO donor. The WI-38 human lung fibroblast cell line was purchased from ATCC (Manassas, VA, USA). WI-38 cells were maintained in minimum essential media (MEM) media (Sigma-Aldrich, Saint Louis, MO, USA) supplemented with 10% fetal bovine serum (FBS) (Gemini Biosciences, Denver, CO, USA), 1% sodium pyruvate, 1% l- glutamine, and 1% penicillin/streptomycin. The cells were maintained at 37 °C, 5% CO_2_. Freshly drawn never frozen bovine blood was purchased from Lampire Biological Laboratories (Everett, PA, USA).

### 2.2 LNP formulation

The liposome was formulated using 1,2-Distearoyl-sn-glycero-3-phosphocholine and 1,2-Distearoyl-sn-glycero-3-phosphoethanolamine-N-[amino(polyethylene glycol-2000] maleimide lipids (9:1 molar ratio). The process of loading NO into the liposome was performed via the standard thin film hydration method as previously published (27). Briefly, a 2.6 mL NO donor (5, 27) solution [1 mg/mL NO donor in PBS: isopropyl alcohol (9:1 v/v)] was added to a thin lipid film for NO donor encapsulation during liposome formation. A volume of 2 mL of the resulting solution was then suspended in 300 mL of DI water and recirculated in a custom tangential flow filtration system to remove a free NO donor. Encapsulation efficiency (EE) was calculated as the total released NO from a filtered lipid nanoparticle (LNP) solution over that released from a neat NO donor solution × 100. The purified LNPs were stored at 4 °C until they were used in further experiments.

### 2.3 LNP characterization

Liposomes were imaged by negative staining transmission electron microscopy (Tecnai T12 (Thermo Fisher Scientific, Hilsboro Oregon, USA), 120 kV). Liposome aliquots (4.5 µL, 2×) were adsorbed to a carbon film grid, and then 4.5 µL of heavy metal salt uranyl acetate was added. The grid was quickly air-dried during which the heavy metal salt formed an amorphous film embedding the liposomes and imaged to observe liposomes. A liposome solution (100 µL) was added to a quartz cuvette and inserted into the measurement chamber of a Malvern Zetasizer Ultra (The zetasizer at CAES - Malvern Analytical, Westborough, MA, USA). The scattering angle was set to 90° and four runs of measurements per sample were conducted to ensure reproducibility of the collected hydrodynamic diameter and polydispersity index (PDI) data.

### 2.4 Drug release characterization

NO release measurements were conducted by chemiluminescence using a GE 280i NO Analyzer (NOA) as previously published (28). Briefly, real-time NO release from NO-loaded and unloaded liposome samples was quantified by the chemiluminescence method using a GE 280i NOA, GE Instruments, CO. To measure NO release, 100 µL liposome solutions were injected into a 10 mL PBS (pH 5.5 or 7.34) at a temperature of 37 °C inside the NOA reaction vessel. The acidic pH environment effect on liposomal drug release was of interest as injured thrombotic vascular regions rapidly acidify to create an acidic microenvironment to support healing, influencing factors like angiogenesis, protease activity, and immune cell function. Under such acidic environments, NO release from carriers is typically accelerated. Nitrogen was used as a carrier gas to transport NO from the reaction vessel to the chemiluminescence detection chamber, and NO released from the samples was measured as the concentration detected by NOA (ppb or ppm). NO flux from the samples was calculated as the quotient of NO concentration detected by NOA (ppb or ppm) and the NOA calibration factor (mol/ppb × sec), and the calculated surface area (cm^2^) of liposomes per 100 µL sample volume and duration NO detection (s or min).

### 2.5 Platelet aggregation experiment

Pooled human platelet rich plasma (PRP) (3 mL/well) was incubated with equal amounts of PLT activator adenosine diphosphate or fibrinogen-grafted LNPs (w and w/o NO payload) or incubated alone at *n* = 4 replicates in a 24-well plate at 37 °C with gentle agitation for an hour. To ensure thorough mixing, the solution was aspirated and dispensed 6×. Each group was incubated in triplicate. Three 10 µL volumes from each well were then placed in a row on a glass slide and covered with glass slips. Each sandwiched 10 µL volume was imaged using a Floid Imager for ImageJ analysis.

### 2.6 Measurement of platelet aggregation size using ImageJ

An aggregation particle size analysis was performed using ImageJ software (version 1.54g). Fluorescent microscopy images of the particles from all wells (*n* = 4 wells per incubation condition) were imported into ImageJ for the analysis. The measurement process involved image preprocessing where the images were converted to gray scale and adjusted for brightness and contrast to enhance the clarity of the nanoparticle boundaries. The image scale was set by using the scale bar embedded in the optical image. The “Set Scale” option in ImageJ was used, and the scale bar length and the corresponding real-world measurement in micrometers (100 µm) were entered. The “Threshold” tool was applied to distinguish the particles from the background. A suitable thresholding method (manual adjustment) was selected to isolate particles clearly. The “Analyze Particles” function was used to measure particle size. Parameters such as size range (30–infinity microns^2^) and circularity (0.1–5) were set to include only particles of interest (ranging from 183 to 679 particles) and exclude artifacts or debris. The “Display Results” and “Show Outlines” options were selected to visualize the identified particles. The software calculated the particle size (area or equivalent diameter) for each identified nanoparticle. The data were exported to a spreadsheet for further statistical analysis. Measurements were reported as mean particle size ± standard deviation. The accuracy of the particle size measurements was validated by repeating the process with multiple images from independent preparations. This method ensured reliable and reproducible measurements of particle size, enabling quantitative comparisons between different formulations.

### 2.7 Biocompatibility study

WI-38 cells were maintained in MEM media supplemented with 10% FBS, 1% sodium pyruvate, 1% l-glutamine, and 1% penicillin/streptomycin. The cells were maintained in a 12-well plate at 37 °C, 5% CO_2_ after adding anti-PLT LNPs at 50% or 100% MEM media volume in triplicate. Control wells received no anti-PLT LNP. The wells were imaged with the Floid imager and the cells were counted in Image J to quantify LNP effects. A similar Image J image analysis as described above was followed, except that a particle size range of 10–15 μm was applied. Counts were recorded for further analysis.

### 2.8 Whole-blood anticoagulation

After recalcification of the whole blood to normal activated clotting time (ACT) and activated partial thromboplastin time (aPTT) values, a static incubation test for up to 10 min of mixing whole blood with LNPs was performed in triplicate by adding 2% of unloaded LNPs and anti-PLT LNPs each to whole bovine blood (v/v). The anticoagulation effect of the anti-PLT LNPs was also evaluated at 10% v/v (*n* = 4 samples). ACT and aPTT data were recorded using the Hemochron Response (Soma Tech. Int., Bloomfield, CT, USA) and Siemens Sysmex CA-560 system Siemens, Malvern, PA, USA.

### 2.9 Statistics

Data were expressed as mean ± SD (standard deviation of the mean). The results were analyzed by comparing means using one-way ANOVA. Values of *p* < 0.05 were considered statistically significant for all tests.

## 3 Results

### 3.1 Overview and rationale

Thrombosis on artificial surfaces remains a major clinical challenge due to its association with significant morbidity and mortality. Platelet adhesion and activation initiate thrombus formation and drive its propagation through the release of procoagulant proteins and the generation of fibrin on the activated platelet surface. Because platelets play a central role in thrombus maturation, they represent a crucial therapeutic target. This biological rationale supports the development of anti-PLT LNPs designed to mitigate thrombosis on artificial surfaces by delivering NO, a well-characterized inhibitor of platelet activation.

### 3.2 Physicochemical characterization of anti-PLT LNPs

#### 3.2.1 Structural design and composition

**Figure 1:**
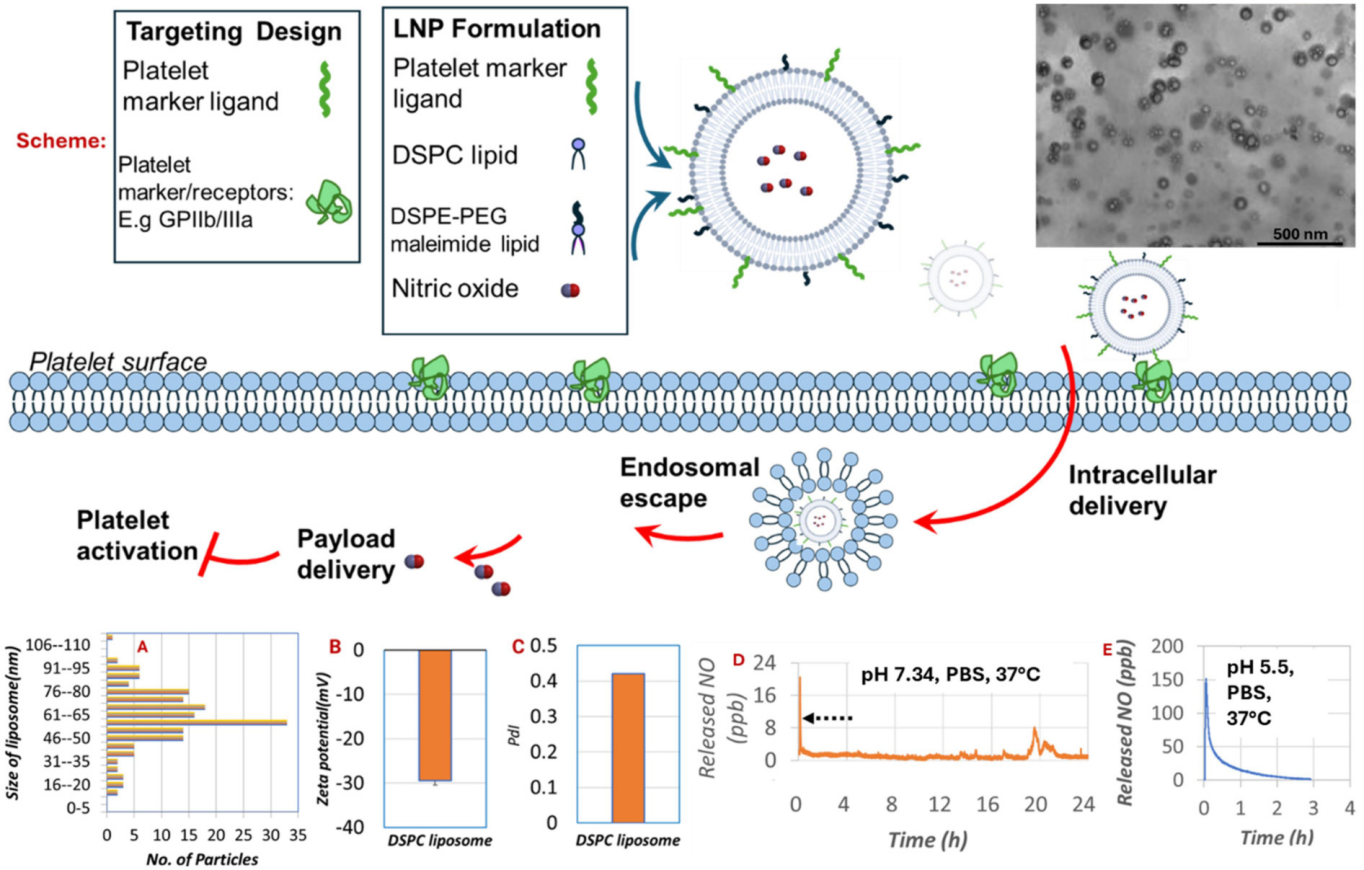
Physicochemical characterization of neutral/PEG LNPs loaded with an antiplatelet drug payload and functionalized with fibrinogen ligand. Design and drug-delivery principles are shown along with a TEM image of the LNPs. The panels include size distribution (**A**), zeta potential (**B**), PDI (**C**), and nitric oxide release profiles under physiological blood pH (**D**) and acidic pH (**E**) conditions.

The design of the NO-loaded anti-PLT LNPs is shown in Figure 1 and incorporates neutral phospholipids, which promote structural stability, effective NO encapsulation, and an antifouling surface, as well as PEG lipids that enhance biocompatibility and allow for controlled NO release. The selection of DSPC and DSPE-PEG maleimide reduces hepatic uptake and renal clearance, supporting prolonged circulation, a key requirement for thrombosis-prevention strategies.

#### 3.2.2 Particle size, zeta potential, and homogeneity

Characterization using transmission electron microscope (TEM), dynamic light scattering (DLS), and NO analysis demonstrated that the LNPs possessed a zeta potential of −29.45 ± 0.07 mV, a polydispersity index of 0.41, and an average diameter of 62.52 ± 9.47 nm (Figures 1A–C). These values indicate a stable and homogeneous nanoparticle suspension with a physiologically favorable size and surface characteristics. Such properties are consistent with reduced macrophage clearance and extended blood residence times, as reported in previous studies (29–33).

#### 3.2.3 NO encapsulation and release profile

An assessment of NO release from 100 µL aliquots of LNPs in PBS at physiological pH (7.34) and acidic pH (5.5) at 37 °C revealed an entrapment efficiency of 49.87% (Figures 1D,E). The NO flux was 79.8 × 10^−5^ mol/min cm^2^ under physiological conditions and increased to 598.4 × 10^−5^ moles/min cm² under acidic conditions, demonstrating a pH-responsive release mechanism. This controlled, stimulus-dependent release profile confirms that the LNPs retain functional NO payload capacity under physiologically relevant conditions and provide sustained antiplatelet activity over time.

### 3.3 Storage stability of LNP formulations

#### 3.3.1 Relevance of long-term stability

Long-term storage stability is critical for the clinical deployment of LNP-based therapeutics. Stable formulations maintain structural integrity, encapsulation efficiency, and functional drug release profiles during storage, which are essential for consistent therapeutic performance, large-scale manufacturing, and distribution.

#### 3.3.2 Fresh vs. stored LNP functionality

**Figure 2:**
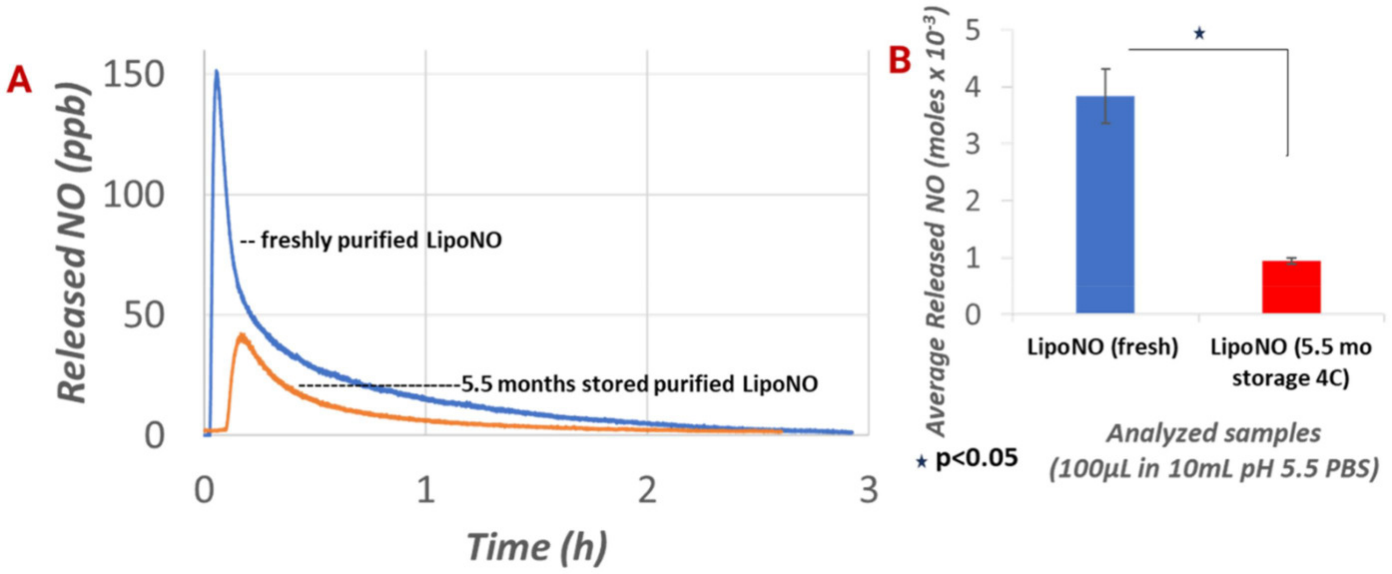
NO release from anti-PLT LNPs measured using GE 280i NOA. Nitric oxide release was measured from a 100 µL of anti-PLT LNP solution reconstituted in a 10 mL PBS at pH 5.5. Freshly purified LipoNO exhibited a higher NO release peak compared with LipoNO stored at 4 °C for over 5 months (**A**) The average total moles of NO released (*n* = 3 per group) also differed significantly between the fresh and the stored formulations (**B**) NOA, nitric oxide analyzer; PBS, phosphate-buffered saline; PLT, platelet.

Comparative NO release analyses revealed that freshly prepared LNPs exhibited approximately threefold higher total NO release compared with LNPs stored at 4 °C (*p* < 0.05) (Figure 2). This suggests the gradual loss of encapsulated NO during storage, likely due to physicochemical instability or increased membrane permeability over time. While the primary objective of the study did not include conducting a full temporal stability assessment, such temporal profiling would provide additional insights.

#### 3.3.3 Strategies to improve storage stability

Potential strategies to enhance stability include the incorporation of cholesterol, which increases membrane rigidity and reduces drug leakage (34), and the application of freeze-drying procedures with cryoprotectants such as dextrose or lactose, which preserve structural integrity and prevent aggregation during dehydration and rehydration (35). Further studies assessing size dynamics, membrane integrity, and functional NO release across storage conditions are warranted to optimize long-term stability.

### 3.4 Antiplatelet activity of NO-loaded LNPs

#### 3.4.1 Inhibition of platelet aggregation

**Figure 3:**
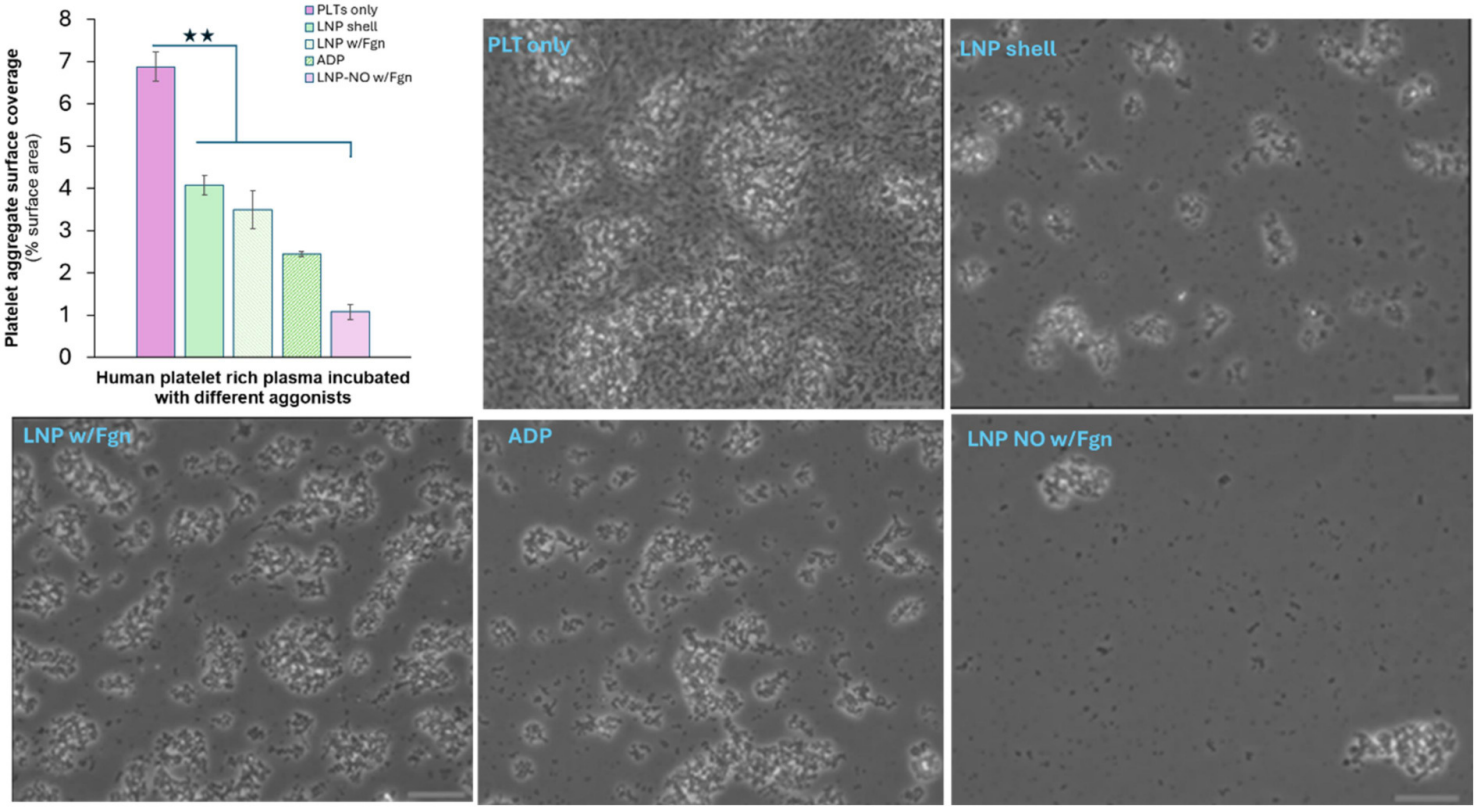
Platelet aggregation in human platelet–rich plasma after 1 h incubation. Platelet-rich plasma was incubated for 1 h in a 96-well plate (37 °C, 35 RPM) under five conditions: no agonist (PLT only), empty liposomes (LNP shell), fibrinogen-grafted LNPs (LNP w/Fgn), ADP, and nitric oxide–loaded, fibrinogen-grafted LNPs (LNP NO w/Fgn). Platelet aggregation was quantified as the percentage of surface area covered by platelet macroaggregates. Scale bar: 100 µm.

The antiplatelet efficacy of the NO-loaded LNPs was assessed by comparing their effects on platelet aggregation with untreated controls and known platelet activators (Figure 3). LNP-treated platelet samples displayed a marked reduction in aggregate formation. Microscopy images corroborated the quantitative data, and ANOVA confirmed statistically significant differences between treatment groups.

#### 3.4.2 Aggregate surface coverage and morphology

Platelets in the untreated control exhibited the most extensive overall aggregation, although the resulting aggregates did not form large clumps. In comparison, the LNP shell, LNP with fibrinogen, and ADP groups developed larger aggregates, with the fibrinogen-containing LNPs producing the greatest aggregate surface area. In contrast, the NO-loaded LNPs with fibrinogen produced the smallest aggregate surface coverage, consistent with effective NO-mediated inhibition of platelet activation. Although additional platelet identity and activation markers (CD41, CD62P) were not evaluated in this study, future analyses incorporating these markers could provide further insight into platelet activation status.

### 3.5 Biocompatibility of anti-PLT LNPs

#### 3.5.1 Importance of biocompatibility assessment

Evaluating LNP biocompatibility is essential to ensure safety in mammalian cell environments and to minimize cytotoxicity or adverse cellular responses. This is particularly important for NO-releasing formulations intended for prolonged blood-contact applications.

#### 3.5.2 Cell viability outcomes

**Figure 4:**
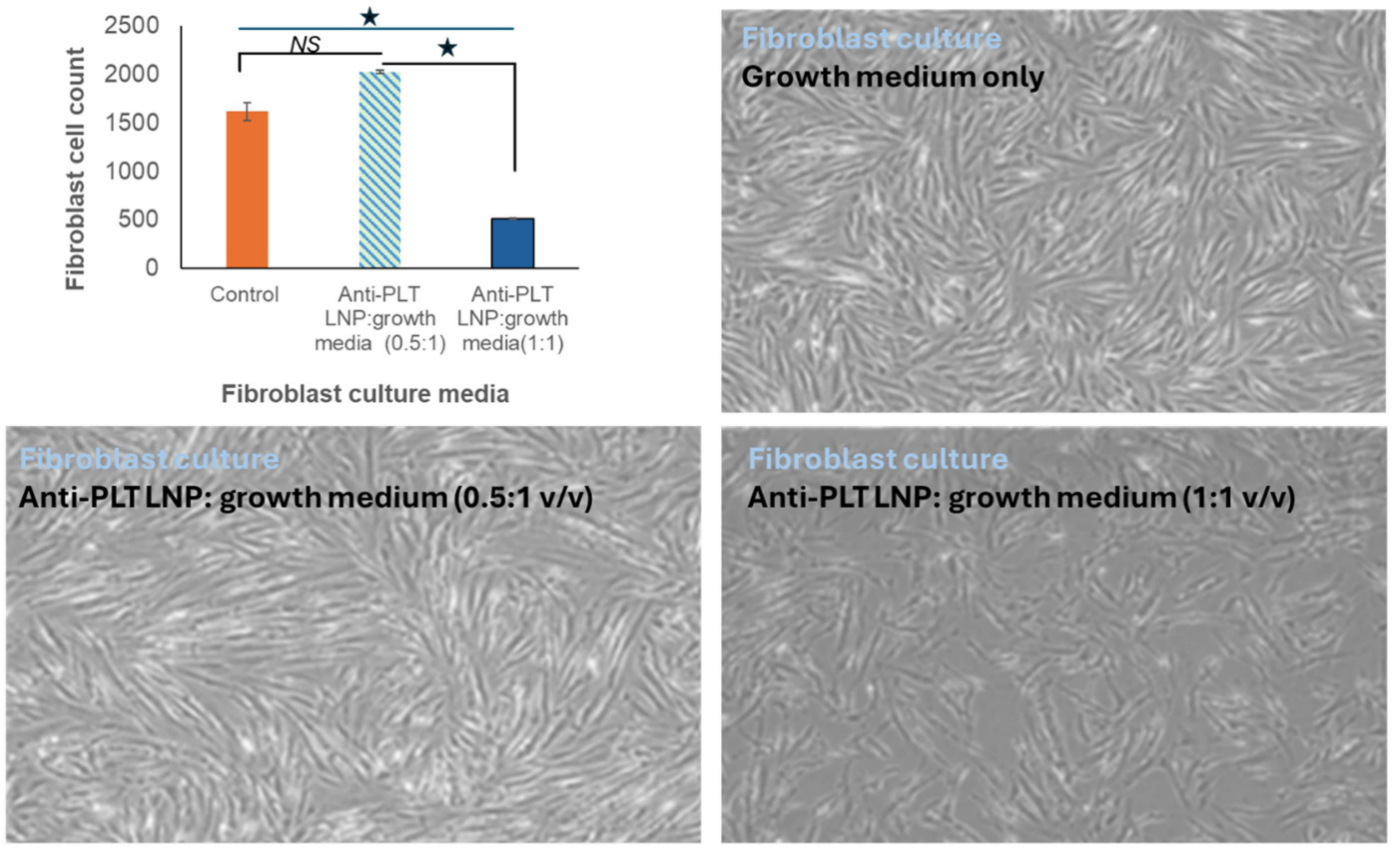
Biocompatibility of anti-PLT LNPs. Light microscopy images of human fibroblasts cultured for 24 h with anti-PLT LNPs mixed with a growth medium at ratios of 0:1 (control), 0.5:1 (9.60 ± 0.09 × 10^−3^ mol NO exposure), and 1:1 (19.20 ± 3.68 × 10^−3^ mol NO exposure) show that cell population density in the 0.5:1 group was comparable to that in the control group. In contrast, a reduction in cell density was observed at the 1:1 ratio, consistent with the known antiproliferative effects of higher NO concentrations.

Fibroblast viability data shown in Figure 4 indicate that low-dose LNP treatment (0.5:1 LNP:medium) did not induce cytotoxicity and resulted in cell counts that exceeded control levels, likely reflecting low-concentration NO-mediated proliferative effects described in the literature (36, 37). At higher doses (1:1), the expected antiproliferative effects of elevated NO concentrations were observed (38), yet overall biocompatibility remained within acceptable limits. These findings support the safety of anti-PLT LNPs across a broad range of therapeutically relevant concentrations.

### 3.6 Effects on blood coagulation parameters

#### 3.6.1 Modulation of ACT and aPTT

**Figure 5:**
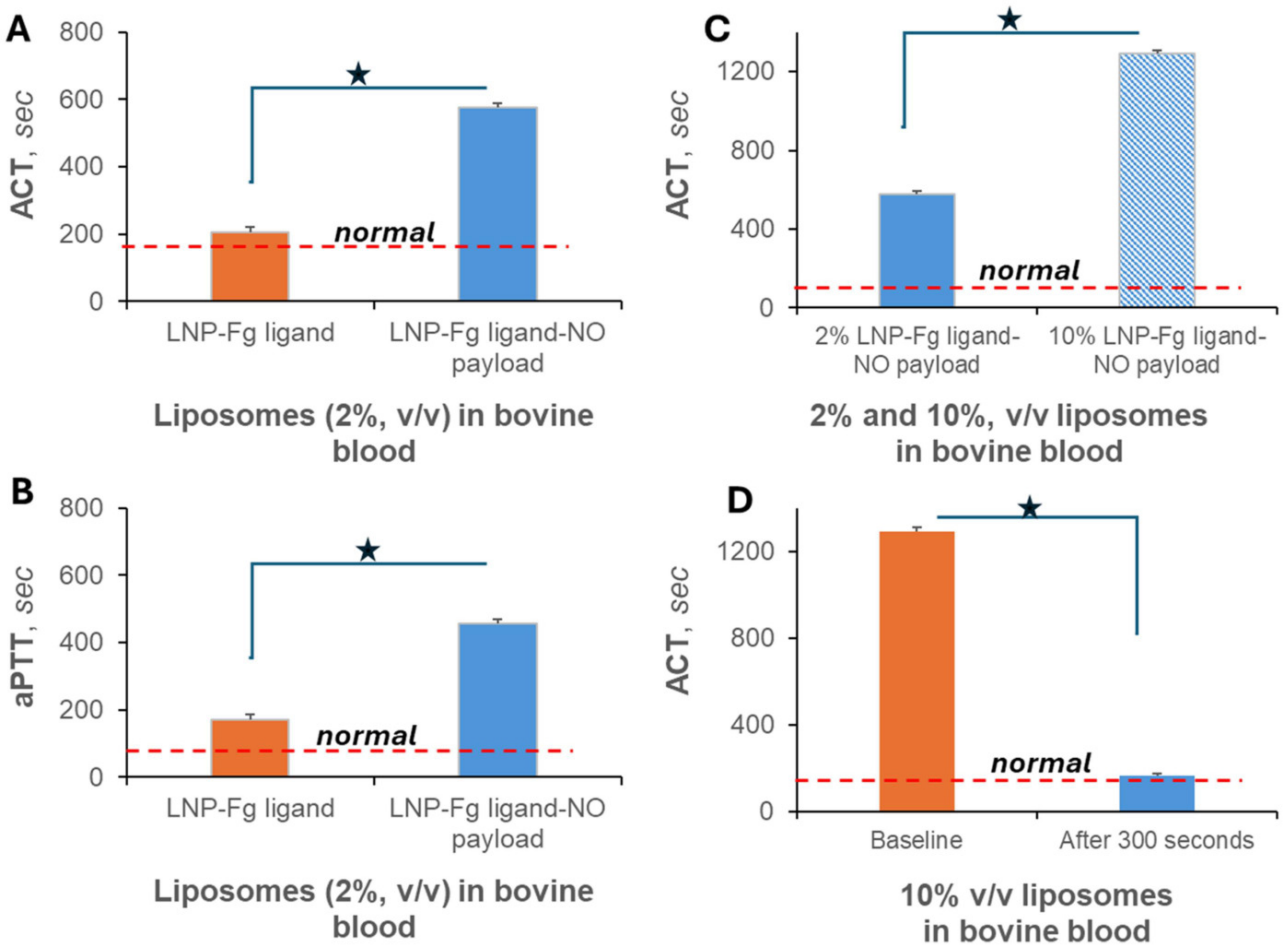
Effect of anti-PLT LNPs on whole-blood coagulation markers. ACT (**A**) and aPTT (**B**) increased following the addition of 2% NO-loaded, fibrinogen-grafted LNPs (v/v). ACT measurements further demonstrated a dose-dependent response, with higher LNP concentrations (2% vs. 10%) producing greater prolongation 1 min after LNP addition (**C**) The sustained effect of 10% LNPs on ACT, measured 300 s post addition, is shown in panel (**D**)**.**

The anticoagulant potential of NO-loaded, fibrinogen-grafted LNPs was evaluated by measuring ACT and aPTT in whole blood (Figure 5). The addition of 2% LNPs significantly increased both ACT and aPTT, indicating effective delay of clot initiation. Increasing the LNP concentration to 10% produced a dose-dependent prolongation of ACT, with sustained effects observed even 300 s after LNP addition.

#### 3.6.2 Implications for blood-contacting medical devices

These findings demonstrate that anti-PLT LNPs exert both antiplatelet and anticoagulant effects and may therefore improve the performance of artificial surfaces used in blood-contacting devices. By reducing thrombosis formation on oxygenators, hemodialysis circuits, and other extracorporeal technologies, these LNPs have the potential to lower device-related failure rates and enhance patient outcomes in clinical settings.

## 4 Discussion

The present study demonstrates the successful formulation, characterization, and functional evaluation of nitric oxide–releasing antiplatelet lipid nanoparticles designed to mitigate thrombosis on artificial surfaces. These findings address a critical need in the field of blood-contacting medical devices, where thrombus formation remains a major barrier to long-term device performance. Platelet-mediated thrombosis is well recognized as the primary initiator of clot formation on biomaterials, driven by rapid platelet adhesion, activation, and aggregation on foreign surfaces (6). Our data align with this established paradigm by showing extensive platelet aggregation in untreated control samples and in groups exposed to prothrombotic stimuli such as fibrinogen or ADP. The strong antiaggregatory effect observed with NO-loaded LNPs reinforces the central role of NO as a potent endogenous inhibitor of platelet activation via cyclic guanosine monophosphate (cGMP)-dependent pathways (39, 40).

The physicochemical properties of the LNPs contributed to their functional performance and therapeutic potential. The observed size, mildly negative surface charge, and moderate PDI are consistent with optimally engineered nanoparticles designed to evade rapid clearance by the mononuclear phagocyte system while maintaining colloidal stability (41, 42). PEGylation has long been known to extend circulation half-life and reduce protein adsorption and opsonization (43–45). The design choices made here, specifically the inclusion of DSPC for membrane rigidity and DSPE-PEG for hydrophilicity, align with previous LNP formulations that demonstrate prolonged systemic residence and reduced immunogenicity (46, 47). These structural features likely contributed to the stable NO retention and controlled release observed in our NO flux studies.

The pH-responsive NO release profile further confirms the functional precision of the LNP formulation. Increased NO release at acidic pH is consistent with that of prior studies showing that acid-labile NO donors or acid-sensitive LNP matrices can enhance NO liberation in microenvironments associated with platelet activation or inflamed tissue (48, 49). Physiologically, activated platelets and developing thrombi can produce localized acidic microenvironments, promoting targeted NO release at the site of thrombosis (50). Thus, the observed pH-dependent release behavior provides a mechanistic advantage over conventional systemic NO donors, which suffer from short half-life and off-target effects (51).

The stability studies revealed a reduction in NO release in stored LNPs relative to freshly prepared formulations. This finding is consistent with reports that NO is prone to diffusion or degradation during prolonged storage, especially in lipid bilayers lacking sufficient rigidity or protective excipients (52). Our results align with prior work showing improved stability when cholesterol is incorporated into liposomal membranes to reduce permeability (53, 54) and represents a logical next step to enhance storage stability for translational applications.

Functional assays provided compelling evidence of the therapeutic potential of the NO-loaded LNPs. Platelet aggregation studies demonstrated significant inhibition of platelet clumping, consistent with earlier reports that LNP-mediated NO delivery can attenuate platelet activation by suppressing intracellular calcium mobilization and integrin αIIbβ3 activation (55, 56). The pronounced reduction in aggregate surface area in the NO-LNP groups compared with fibrinogen- or ADP-stimulated controls mirrors findings from studies in which NO donors prevent the transition from platelet adhesion to stable aggregation (57, 58). Although platelet activation markers such as CD41 and CD62P were not assessed in this study, their inclusion in future work would strengthen mechanistic conclusions.

Biocompatibility results demonstrated that anti-PLT LNPs are safe across a range of concentrations, with low-dose formulations even promoting fibroblast proliferation. This is consistent with evidence that low-level NO stimulates cell growth, migration, and collagen synthesis (59, 60). Conversely, the antiproliferative effect observed at higher concentrations aligns with the well-documented biphasic role of NO, in which elevated levels inhibit mitochondrial respiration and induce cytostasis (61, 62). These findings confirm that the LNP-delivered NO behaves within known physiological boundaries and supports the safety of the formulation for eventual *in vivo* use.

The anticoagulant effects observed in whole blood further strengthen the translational relevance of the system. Prolonged ACT and aPTT values following treatment with NO-loaded, fibrinogen-grafted LNPs are consistent with NO-mediated inhibition of thrombin generation and reduced fibrin formation, as previously described in NO-based anticoagulant research (63, 64). The dose-dependent and sustained prolongation of ACT at higher LNP concentrations suggests that the system is capable of producing long-lasting anticoagulation, an essential property for application in extracorporeal circuits, vascular grafts, and artificial lungs, all of which are highly prone to thrombosis (65, 66).

**Figure 6:**
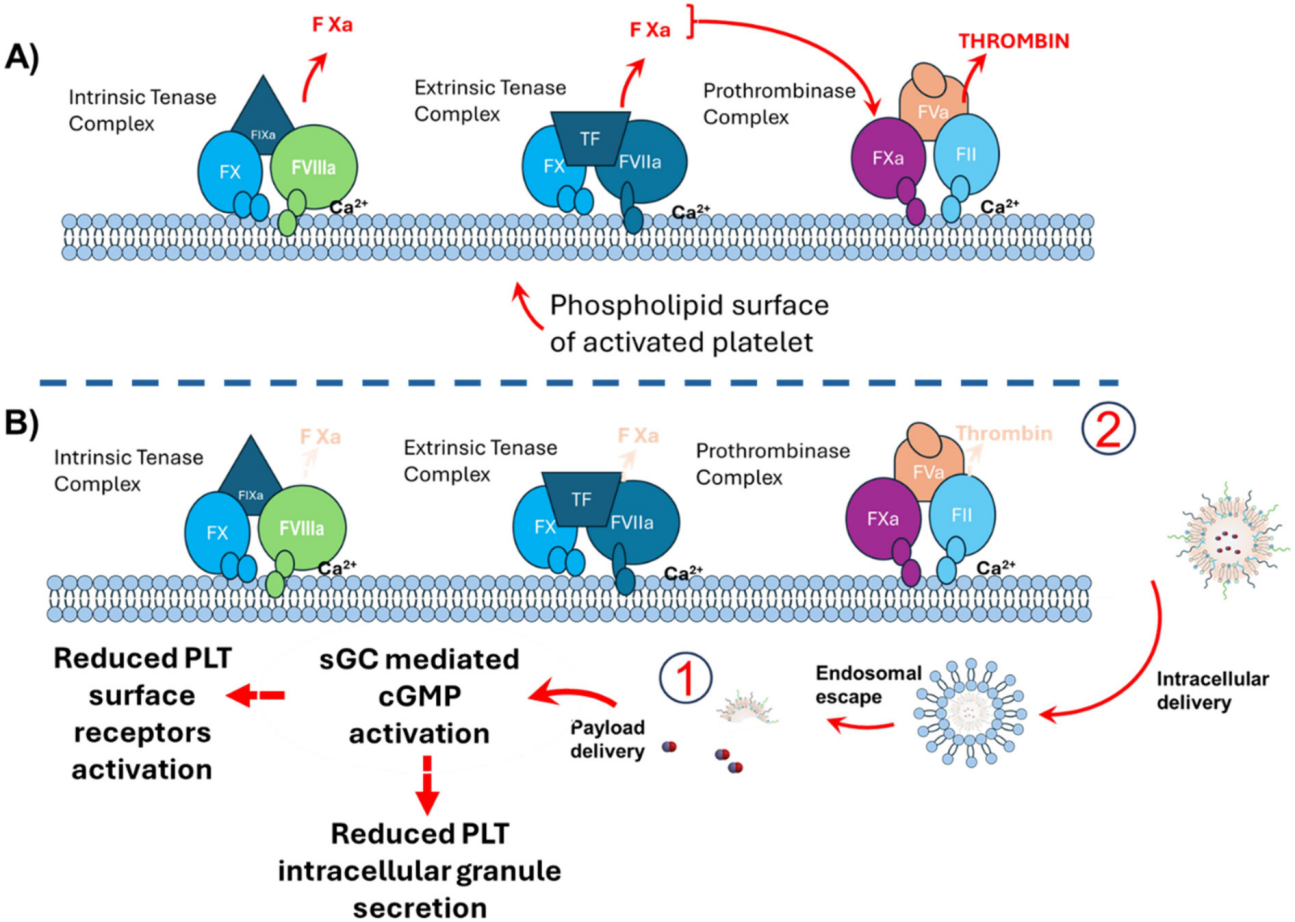
Proposed mechanism of action of anti-PLT LNPs. Clot propagation relies on the activation of factor X and the generation of thrombin on the phospholipid surface of activated platelets (**A**), where assembly of the tenase and prothrombinase complexes occurs. Delivery of NO from anti-PLT LNPs is proposed to inhibit platelet activation through soluble guanylyl cyclase (sGC)–mediated cGMP activation, thereby limiting the formation of these coagulation complexes and subsequent fibrin production (**B**) As platelet activation reduces, the assembly of procoagulant complexes on the platelet surface diminishes, ultimately leading to decreased clot formation.

We illustrate the proposed mechanism of action for anti-PLT LNPs in reducing coagulation (Figure 6). During clot formation, the activation of clotting factor X and the release of thrombin from platelets are supported by the phospholipid surface of activated platelets, which assembles the protein complexes necessary for these processes to occur (top panel). Since the anti-PLT LNPs were designed to deliver nitric oxide to inhibit platelet activation, it is thought that a reduction in platelet activation will reduce the assembly of the tenase and prothrombinase complexes, as well as fibrin formation on the platelet surface (bottom panel). A diminished activation is expected to disrupt the cascade of events leading to clot propagation, resulting in reduced thrombus formation. This mechanistic insight underscores the potential of anti-PLT LNPs to effectively target and mitigate platelet-driven thrombotic processes.

## 5 Conclusion

Taken together, the results of this study provide compelling evidence that NO-loaded anti-PLT LNPs represent a promising strategy for reducing thrombosis on artificial surfaces. Their physicochemical stability, controlled NO release, strong antiplatelet effects, and favorable biocompatibility profile position them as a viable platform for translation into clinical settings. Despite these promising findings, the study is limited by its reliance on *in vitro* models and the absence of long-term stability and mechanistic assessment gaps (66–73). In addition, manufacturability and scalability of the anti-PLT LNPs would require high throughput and scalable formulation technology. Future work will focus on optimizing formulation stability (e.g., cholesterol incorporation and cryoprotective lyophilization), integrating these LNPs into extracorporeal membrane oxygenation or stent coatings for dynamic flow testing, validating efficacy under dynamic flow and *in vivo* thrombosis models, incorporating mechanistic platelet activation assays, and studying rapid formulation technologies. With continued refinement, these LNPs could significantly improve the hemocompatibility and functional lifetime of a broad range of medical devices, optimizing long-term storage stability.

## Data Availability

The datasets presented in this article are not publicly available due to commercial confidentiality and proprietary restrictions. Requests to access the data should be directed to corresponding author, subject to approval by the data owner.
