# Supplementary material for: Drug-loaded nanoparticles reduced platelet aggregation and blood coagulation
Source: Front Med Technol. 2026 Feb 4;7:1690389. doi: 10.3389/fmedt.2025.1690389 (PMC12913426; doi:10.3389/fmedt.2025.1690389)
Supplement: Supplementary file 2 [file Datasheet2.pdf]

**Drug-loaded Nanoparticles Reduced Platelet Aggregation and Blood Coagulation**

[1]Sarah Majin MSc Eng., [1]Afrida Malik MSc Eng., [1]Pratima Poudel MSc Eng. [2]Vince Faustino M.D., [2]Nubia Zuverza-Mena Ph.D., [2]John Hwa M.D. Ph.D., [2]Seyedtaghi Takyar M.D Ph.D [3]Susan Shea Ph.D., [1]Kagya Amoako Ph.D.

**Corresponding Author:**

Kagya Amoako PhD, kamoako@newhaven.edu

**Affiliations**

[1]Department of Chemistry and Chemical & Biomedical Engineering, University of New Haven, West Haven CT.

[2]Yale University, New Haven CT.,

[3]University of Pittsburgh, Pittsburgh PA.

**Keywords**

Platelets, lipid nanoparticles, Thrombosis, Medical Devices, Artificial Surfaces.

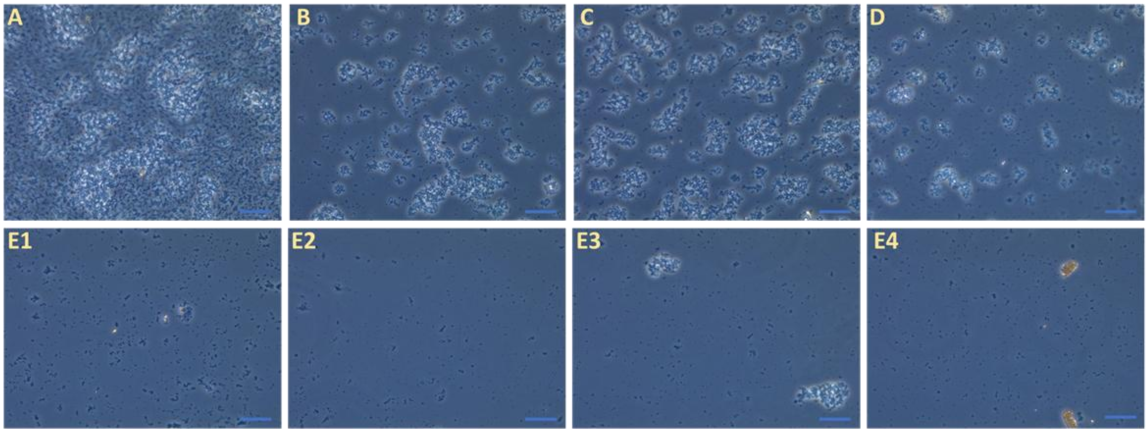


**Fluorescent microscopy of platelet aggregation** from human platelet rich plasma incubated for 1 hour in a 96 well plate (37$^{o}$C, agitation at 37 RPM) without agonist (A), and with ADP agonist interaction (B), empty liposome construct interaction (C), fibrinogen-grafted empty liposome construct interaction (D), and nitric oxide encapsulated fibrinogen-grafted liposome construct interaction (E1-4) Scale bar (100 *µm*).

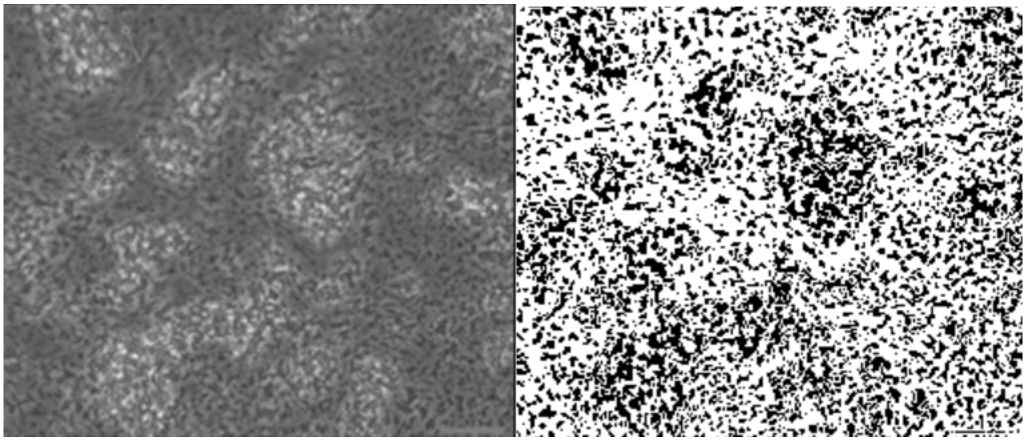

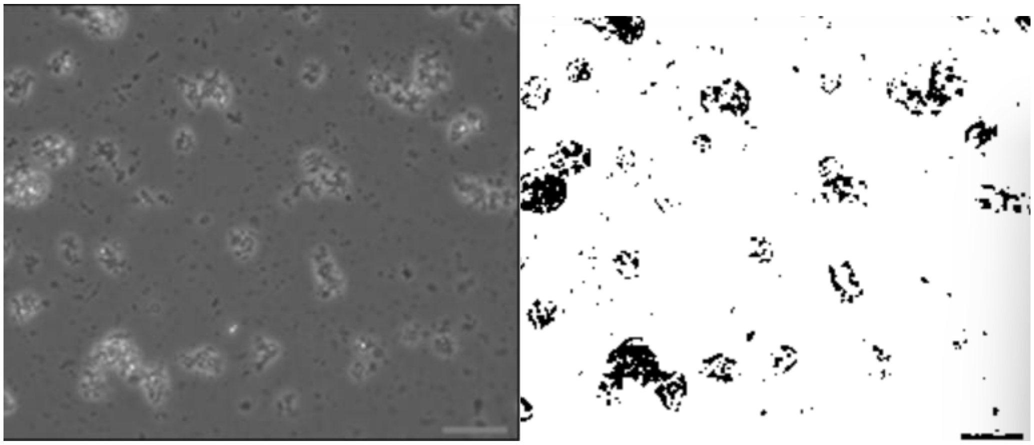

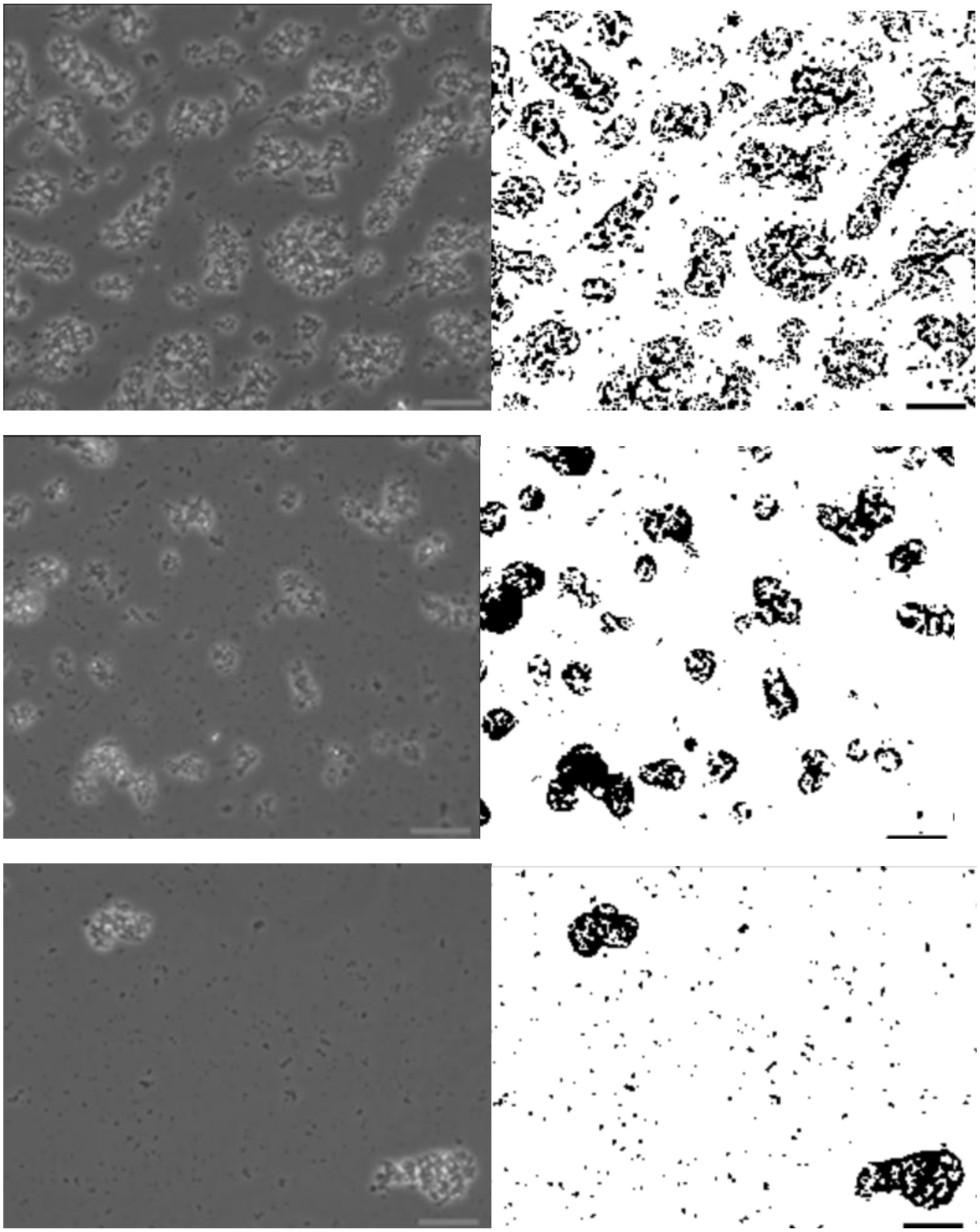

**Image conversion using ImageJ for particle analyses of platelet aggregation assessment.**
