# Supplementary material for: Drug-loaded nanoparticles reduced platelet aggregation and blood coagulation
Source: Front Med Technol. 2026 Feb 4;7:1690389. doi: 10.3389/fmedt.2025.1690389 (PMC12913426; doi:10.3389/fmedt.2025.1690389)
Supplement: Supplementary file 1 [file Datasheet1.pdf]

Kagya Amoako Ph.D.
University of New Haven
kamoako@newhaven.edu
8-19-2025

Dr. Elena Del Favero
Editor-in-Chief, *Frontiers of Medical Technology*

Subject: Manuscript Submission – Drug-loaded Nanoparticles Reduced Platelet Aggregation and Blood Coagulation

Dear Dr. Del Favero,

We are very pleased to submit our research manuscript to *Frontiers* following the recent publication of our review article also in the *Frontiers* journal. It is an honor to contribute to a journal that plays a pivotal role in advancing biomaterials and macromolecular therapeutics.

Our study addresses the pressing clinical challenge of surface-induced thrombosis, which accounts for a substantial portion of medical device-related complications. We present anti-platelet lipid nanoparticles (anti-PLT LNPs) formulated with nitric oxide cargo, demonstrating prolonged stability, biocompatibility, and potent inhibition of platelet aggregation and blood coagulation. These findings underscore the potential of anti-PLT LNPs as a promising platform for targeted platelet therapies.

We believe this work aligns well with the journal's scope and will be of significant interest to researchers in nanomedicine, biomaterials, drug delivery, and cardiovascular medicine. We look forward to your consideration and are happy to provide any additional information.

Thank you for your time and consideration.

Sincerely,

Kagya Amoako Ph.D.
